# The mediating effect of parenting style on the relationship between first-born children’s temperament and psychological adaptation

**DOI:** 10.1038/s41598-022-17897-3

**Published:** 2022-08-10

**Authors:** Guoying Qian, Sijie Yang, Ruonan Li, Gang Dou

**Affiliations:** 1 College of Preschool Education, Capital Normal University, Beijing, China; 2 Heping Street Kindergarten of Chaoyang District, Beijing, China; 3 School of Education, Hubei University of Arts and Science, 296 Longzhong Road, Xiangyang, Hubei China

**Keywords:** Human behaviour, Psychiatric disorders, Health policy, Public health, Risk factors, Psychology and behaviour

## Abstract

Chinese first-born children need to learn how to get along with their siblings after the implementation of the universal two-child policy in 2016. We investigated the relationships between temperament, parenting style, and psychological adjustment among firstborns. The current study employed a questionnaire survey conducted in four regions in China. A total of 524 Chinese two-child families participated in the study; the firstborns were between 3 and 8 years old and their younger siblings were between 1 month and 5 years old. The results indicated that (1) children’s temperament subscales were significantly related to parenting style subscales and psychological adaptation. Moreover, the parenting style subscales were significantly related to psychological adaptation, and (2) authoritarian parenting partially mediated the relationship between approach or withdrawal and psychological adjustment.

## Introduction

On January 1, 2016, China ended the one-child policy that had been implemented for more than 30 years, by allowing couples to have two children. Five years later, in 2021, China further relaxed this limitation and now permits three children per couple. However, statistical data and policy evaluation studies showed that adjustments in family planning policies had not led to a sustained increase in the number of births^1^. Some researchers believed that the reason can be partly attributed to the decline in fertility willingness of Chinese couples^2^.

The transition from one-child to two-child families has led to increasing economic burdens and restructuring of relationships within families. Most preschoolers also changed from single children to first-born children. For the latter, who are accustomed to being the only child, the second or third child’s arrival causes adjustment difficulties. Negative news reports about the first-born child in two- or three-child families are also increasing^3^. First-born children’s psychological adaptation to siblings has received significant attention in Chinese society and academic circles. Psychological adaptation is the process by which individuals adjust to changes in the external environment to ensure that their covert dispositions and overt behaviors are more consistent with the requirements of environmental changes and their personal development. Hence, through this process, the subject and the environment achieve a new balance^4^. In this study, psychological adaptation during the transitional period is mainly manifested in emotion and behavior.

Changes in family relationships and environment after the birth of a second child may result in diverse reactions in first-born children. There are individual differences in whether firstborns experience distress after the birth of their siblings^5^. Some children experience distress and exhibit destructive behaviors^6^ such as jealousy, grumpiness, and aggressive tendencies. However, some react positively^7,8^. They might hug, offer to help, and even kiss the newborns. Most children’s reactions tend to be a combination of these two.

Children’s temperament is an important individual difference^9^. Firstborns with difficult temperaments (highly active and emotionally intense) are reluctant to accept their younger siblings^10–13^. Children of different temperaments respond differently. For instance, those with difficult temperaments who protest when their mothers look after their 14-month-old younger siblings, often feel negative emotions such as anger^7,14^. They could exhibit a propensity for emotional disorders or maladaptive problems^15–17^, and often show negative emotions (e.g., anger), lack of adjustment (e.g., clinginess), and poor emotional management skills (e.g., crying), affecting their response to the second-born child^18,19^ and demonstrating less pro-social behavior^20^. First-born children with difficult temperaments often resist their siblings’ birth, and show poor social adaptation and social interaction skills^7^ along with difficulty in establishing positive interpersonal relations^12^. For example, previous studies have suggested that first-born children with difficult temperaments demonstrate more social deviations and sleep problems during the transition period, and are less willing to take care of their siblings^14^. Dunn found that those with more negative emotions had more social withdrawal, insecure attachment, and sleeplessness, and were unwilling to help after the second child’s birth^21^. A study suggested that the second child’s arrival and the change in the first child’s identity may prompt a shift in the first child’s temperament type to a difficult one^3^.

Previous studies have found that the activity levels and emotional aspects of temperament impact first-born children’s adaptation^13,22^. How do approach/withdrawal and rhythmicity, which have a relatively stable temperament dimension in infants and young children, affect firstborns’ psychological adjustment? Approach or withdrawal refers to children tending to approach or avoid the new environment, and rhythmicity reflects the regularity of their diet and daily life. Research suggests that children who enjoy the new environment tend to be more socially competent^23^; those with relatively regular living habits are more likely to adapt well to newborns^24^.

Along with temperamental characteristics, parenting styles, which reflect parental beliefs, attitudes^25^, and behaviors^26^ while raising their children^27^ are important during the change in firstborns’ identity. An appropriate parenting style can reasonably coordinate sibling relationships and promote a smooth transition. There is a strong connection between parenting styles and children’s problematic behaviors^28^. Firstborns show problematic behavior when their parents make excessive demands^29^. Tan et al. found that parents with greater pressure were more inclined to choose authoritarian parenting styles, leading to an increase in covert and overt behavioral difficulties^30^ and adversely influencing children’s personality development and their ability to understand and explore novelty in their surroundings^31^.

Thus, on the one hand, children with difficult temperament are more likely to exhibit problematic behaviors after the birth of their younger siblings^32^. On the other hand, temperament types may affect the problematic behaviors of firstborns through parenting styles. Due to the difficult temperament of the first born children, their parents may be more likely to adopt an authoritarian parenting style without a better choice, which may also lead to more problematic behaviors in firstborns^12,33^. Therefore, it is reasonable to believe that parenting style may mediate the relationship between children's temperaments and problematic behaviors.

While current researches have focused on maternal parenting behaviors such as punishment, excessive control, and overprotection^34^, less attentions have been paid to the parenting style of non-one-child families and other aspects of firstborns’ psychological adjustment. Among first-born children, emotionality and activity receive more attention, while approach or withdrawal and rhythmic temperament receive less focus. Specifically, there are few studies on first-born children in mainland China. Therefore, this study explored the relationship among parenting style and firstborns’ temperament and adjustment adaptation in China.

Temperament, which plays a unique role in children's development independent of the environment, interacts with environmental factors to jointly affect childhood development and adaptation^35^. Volling proposed a developmental ecological systems (DES) model to examine the change in family and children’s functioning during the transition to siblinghood. In the DES model, children are placed in the ecological environment and the system indicates that the internal and external systems of the family are important for children’s adaptation. Children grow up in a dynamic and constantly changing environment. The DES model analyzes their environment across the following levels: (a) parents’ psychological characteristics, e.g., their well-being and personality characteristics; (b) children’s characteristics, e.g., temperament; (c) family environment (microsystem); (d) the larger social environment wherein the family is located. Each system influences children’s development and adaptation through their interaction^36^.

Based on the DES model and the framework of the Belsky model^32^, Feinberg^37^ proposed the co-parenting ecological systems, which discussed the relationship between the family system and children's developmental adaptation regarding three aspects: individual, family, and environment. Children's characteristics affect their adaptation directly through the mediating effect of co-parenting.

**Figure 1:**
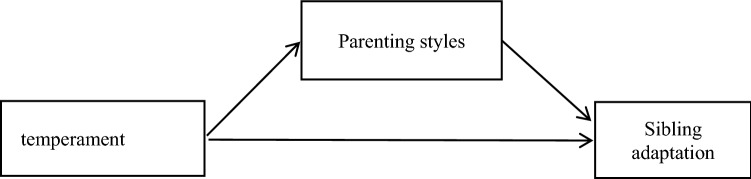
Mediation model.

Based on these previously discussed studies and theories, we hypothesize that first-born children’s adjustment adaptation will be directly affected by their temperament and parental style, and temperament will also affect first-born children's adaptation through the parenting style (see Fig. 1)

.

## Method

### Participants

The study was approved by the Research Ethics Committee of the first author's institution. A total of 600 two-child families were randomly recruited from Shanxi, Shaanxi, Hainan and Shanghai . The questionnaire was completed by the mothers of the children. After removing 76 families whose questionnaires were incomplete, 524 families finally entered the study. There were 261 male and 263 female firstborns ranging from three to eight years old (*M* = 6.00, *SD* = 1.62) and their younger siblings (251 male and 273 female), ranging from one month to five years old (*M* = 3.30, *SD* = 1.84). The age difference between firstborns and their younger siblings was three years. 70% of families (n = 368) had a combined income of over $10,000, and 60% of parents had a college degree or above (n = 314).

### Psychological adaptation

The First-Born Behavior Questionnaire was used to evaluate firstborns’ psychological adjustment. It was developed from the Child’s Behavior Questionnaire (Mother Assessment)^38^ and comprises 18 questions in five subscales^39^. The subscales’ Cronbach α varies from 0.70 to 0.82*.* The higher the score in the *First-Born Behavior Questionnaire*, the worse the psychological adjustment.

### Parenting style

The Parenting Style Questionnaire^40^ comprises 40 items across five subscales: spoiling parenting (e.g., “Buy what the first-born children want”; Cronbach’s α = 0.81); democratic parenting (e.g., “Encourage the first-born children to do what they want to do”; Cronbach’s α = 0.71); indulging parenting (e.g., “Not caring about first-born children's wants and desires”; Cronbach’s α = 0.84), authoritarian parenting (e.g., “Beat or scold the first-born children when they disobey their parents”; Cronbach’s α = 0.66); disaccording parenting (e.g., “Sometimes meet the first-born children’s unreasonable demands, and sometimes reject them”; Cronbach’s α = 0.82). Participants responded from one (never) to five (always). Overall, Cronbach’s α was 0.82. The higher the score, the more prominent the type in that dimension. Firstborns’ parents were asked to fill the questionnaire according to their present situation.

### Temperament scale

The *Temperament Questionnaire*^24,41^ comprises 72 items across nine subscales, among which four were used in this study: (1) activity level (e.g., “Active, unable to settle down, and constantly runs, climbs up and down, or twists the body when playing in the amusement park”; Cronbach’s α = 0.62); (2) quality of mood (e.g., “Looks significantly happy when playing with other children”; Cronbach’s α = 0.61); (3) rhythmicity (e.g., “Defecates regularly every day”; Cronbach’s α = 0.65); (4) approach/withdrawal (e.g., “Likes to try new foods”; Cronbach’s α = 0.60). Participants respond on a 7-point scale ranging from one (never) to seven (always). A higher score on each dimension represents higher speed and frequency of physical activity, more friendliness, more regularity in eating and living habits, and reduced distance from the new environment.

### Ethics declaration

The studies involving human participants were reviewed and approved by the Scientific Research Ethics Committee of College of Preschool Education, Capital Normal University. All procedures performed in studies involving human participants were in accordance with the ethical standards of the institutional research committee and with the 1964 Helsinki Declaration and its later amendments. The participants provided their written informed consent to participate in this study.

## Results

Pearson’s correlation analysis was used to examine relations among first-born children’s temperament, parenting style, and psychological adaptation. Psychological adaptation was positively related to activity level (*r* = 0.12, *p* < 0.001), spoiling parenting (*r* = 0.16, *p* < 0.001), indulging parenting (*r* = 0.19, *p* < 0.001), authoritarian parenting (*r* = 0.24, *p* < 0.001), and disaccording parenting (*r* = 0.22, *p* < 0.001). It was negatively related to approach or withdrawal (*r* = − 0.15, *p* < 0.001), quality of mood (*r* = − 0.09, *p* < 0.05), and democratic parenting (*r* = − 0.14, *p* < 0.001). Activity level was positively related to spoiling parenting (*r* = 0.11, *p* < 0.001), indulging parenting (*r* = 0.17, *p* < 0.001), authoritarian parenting (*r* = 0.05, *p* < 0.05), and disaccording parenting (*r* = 0.19, *p* < 0.001). It was negatively related to democratic parenting (*r* = − 0.22, *p* < 0.001). Rhythmicity was positively related to democratic parenting (*r* = 0.13, *p* < 0.001) but negatively related to indulging parenting (*r* = − 0.13, *p* < 0.001), authoritarian parenting (*r* = − 0.10, *p* < 0.05), and disaccording parenting (*r* = − 0.17, *p* < 0.001). Approach or withdrawal was positively related to democratic parenting (*r* = 0.15, *p* < 0.001) but negatively related with spoiling parenting (*r* = − 0.18, *p* < 0.001), indulging parenting (*r* = − 0.22, *p* < 0.001), authoritarian parenting (*r* = − 0.13, *p* < 0.001), and disaccording parenting (*r* = − 0.13, *p* < 0.001). Quality of mood was positively related to democratic parenting (*r* = 0.25, *p* < 0.001) but negatively related to spoiling parenting (*r* = − 0.14, *p* < 0.001), indulging parenting (*r* = − 0.20, *p* < 0.001), authoritarian parenting (*r* = − 0.14, *p* < 0.001), and disaccording parenting (*r* = − 0.21, *p* < 0.001) (see Table 1).

**Table 1:** Pearson’s correlation coefficients of the study variables.

	*M* ± *SD*	1	2	3	4	5	6	7	8	9	10
1	3.94 ± .85	–									
2	5.15 ± .89	0.12**	–								
3	4.61 ± .71	− 0.08	− 0.20**	–							
4	1.81 ± .60	− 0.15**	− 0.04	0.18**	–						
5	4.96 ± .66	− 0.09*	− 0.24**	0.25**	0.31**	–					
6	4.02 ± .52	0.16**	0.11**	− 0.07	− 0.18**	− 0.14**	–				
7	2.09 ± .61	− 0.14**	− 0.22**	0.13**	0.15**	0.25**	− 0.18**	–			
8	2.62 ± .55	0.19**	0.17**	− 0.13**	− 0.22**	− 0.20**	0.51**	− 0.36**	–		
9	2.39 ± .74	0.24**	0.05*	− 0.10*	− 0.13**	− 0.14**	0.37**	− 0.24**	0.44**	–	
10	2.64 ± .48	0.22**	0.19**	− 0.17**	− 0.13**	− 0.21**	0.41**	− 0.24**	0.53**	0.54**	–

1, psychological adaptation; 2, activity level; 3, rhythmicity; 4, approach/withdrawal; 5, quality of mood; 6, spoiling parenting; 7, democratic parenting; 8, indulging parenting; 9, authoritarian parenting; 10, disaccording parenting.

*p < 0.05, **p < 0.01.

A hierarchical multiple regression analysis was used to examine the predictability of first-born children’s age, gender, age difference, family income, parenting style, and temperament on psychological adjustment. The results in Table 2 show that age, gender, age difference, and family economic income were added to the first layer. For demographic variables such as economic income, the predictive variable model was significant, F <4519>  = 5.89, p < 0.01. These demographic variables could significantly predict first-born children’s psychological adjustment (p = 0.000) with 5% variance, specifically regarding age difference and family income. The second layer added the dimensions of temperament and parenting style, and the predictive variable model was significant, F <10,523>  = 6.91, p < 0.01. After controlling for the aforementioned demographic variables, approach/withdrawal and authoritarian parenting could significantly predict psychological adaptation (t = − 2.06, β = − 0.09, *p* < 0.05; t = 2.48, β = 0.13, *p* < 0.05) with 11% variance.

**Table 2:** A hierarchical multiple regression analysis.

	*B*	*β*	*t*	*F*	*R*^*2*^	*△R*^*2*^
**The first layer**				14.16**	0.05	0.05
Constant	3.02					
Family economic income	− 0.087	− 0.17	− 4.07**			
Age difference	− 0.033	− 0.15	− 3.51**			
**The second layer**				6.91**	0.13	0.11
Constant	2.75					
Activity level	0.04	0.08	1.73			
Rhythmicity	− 0.01	− 0.02	− .34			
Approach/withdrawal	− 0.06	− 0.09	− 2.06*			
Quality of mood	− 0.01	− 0.10	− 0.21			
Spoiling parenting	0.04	0.05	1.00			
Democratic parenting	− 0.02	− 0.02	− 0.48			
Indulging parenting	0.00	0.00	− 0.01			
Authoritarian parenting	0.10	0.13	2.48*			
Disaccording parenting	0.05	0.08	1.52			

*p < 0.05, **p < 0.01.

Model 4 assessed the influence of authoritarian parenting on the relationship between approach/withdrawal and psychological adaptation, controlling age difference and family income (see in Table 3). The results showed that there was a significant direct path from approach/withdrawal to psychological adaptation (*β* = 0.09, *p* < 0.01) in the absence of authoritarian parenting. Approach/withdrawal could significantly predict authoritarian parenting (*β* = − 0.09, *p* < 0.01). Psychological adjustment was significantly associated with approach/withdrawal (*β* = 0.07, *p* < 0.01) and authoritarian parenting (*β* = − 0.16, *p* < 0.01). Therefore, authoritarian parenting partially mediated the relationship between approach or withdrawal and psychological adaptation.

**Table 3:** Testing the mediation effect of authoritarian parenting.

Predictors (IV)	Model 1 DV: psychological adaptation		Model 2 DV: authoritarian parenting		Model 3 DV: psychological adaptation	
	*β*	*t*	*β*	*t*	*β*	*t*
Approach/withdrawal	− 0.09	− 3.25**	− 0.09	− 2.69**	− 0.07	− 2.74**
**Authoritarian**					0.16	4.72**
R^2^	0.07		0.03		0.11	
F	13.13**		6.03**		15.82**	

*p < 0.05, **p < 0.01.

A bootstrap procedure evaluated the size of the indirect effect and confidence intervals. The indirect effects of approach or withdrawal on psychological adaptation, mediated by authoritarian parenting (ab = − 0.02, *SE* = 0.01, 95% CI [− 0.03, − 0.01]), was significant. The total effect accounted for 17.31% by the mediation effect. Zero was not included in the 95% confidence interval, showing that approach or withdrawal had a significant indirect effect on psychological adaptation through authoritarian parenting.

## Discussion

Relationships among temperament, parenting style, and psychological adaptation of first-born children were examined. First, highly active or emotionally intense temperaments were positively associated with psychological maladaptation, which was consistent with some previous findings in Western literature^7,8,12,14–19,21^. We also found that approach/withdrawal predicted psychological adjustment, which suggested that first-born children who willingly approached new environments could adapt to a younger sibling’s birth, while those who avoided new environments exhibited maladaptation. This finding is similar to those of previous studies on Chinese only children in kindergarten, whose approach/ withdrawal predicted their psychological and physical changes^42^, and also predicted the teacher-student relationship quality two years later^43^.

First-born children who tended to approach new environments are more likely to adapt well to their new siblings. The second-born child’s birth did not cause pressure, and they did not need to mobilize their psychological resources to cope, which reduced the degree to which they perceived this stressful event as a potential threat. However, young children generally avoid new environments due to the fear of the changes in the family^44^. This can be explained by Family Stress Theory, which stated that a second-born child’s birth is perceived as a life-stressing event for first-born children and their families, presenting as an inevitable psychological threat to each family member^21,34,39^. Therefore, it was normal for first-born children to show a significant increase in behavioral problems and psychological discomfort during the transition period. If first-born children changed their attitudes toward the second-born children, the stress would be reduced, benefiting their adaptation^45,46^.

Second, our results suggested that authoritarian parenting partially mediated the relation between approach or withdrawal and psychological adaptation. In one case, the more a child's temperament leans toward withdrawal, the more likely his parents are to adopt an authoritarian parenting style, which may lead to the worse the child's psychological adaptation. This was consistent with previous results that parenting style was an important factor affecting children’s reaction^47^. Parents’ warmth or control were key factors regarding children’s obedience^48^, and different kind of parents played a different role in preschool children’s emotional approach/withdrawal. Another case is, the more a child's temperament leans toward approach, the less likely his parents are to adopt an authoritarian parenting style, which may lead to the better the child's psychological adaptation. And this finding was also consistent with previous studies that children in low-control families were more likely to develop independently^49^, and those with stronger independent abilities would not rely excessively on other people^43^; hence, they could adapt better.

According to Volling’s^36^ DES and Feinberg’s^37^ co-parenting ecological models, children's and family’s characteristics have a combined effect on their adaptation. Approach/withdrawal temperament was a contributing factor to authoritarian parenting. The more reluctant the firstborns were in accepting the secondborns during the transition period, the more the parents would be arbitrary and demand their children do things according to their standards, ignoring firstborns’ inner feelings, show a lack of patience and enthusiasm, failing to encourage and praise, and require firstborns to obey commands unconditionally. When such conditions prevail, first-born children reject novelty and develop personality traits such as low self-esteem, withdrawal, anxiety, and inhibited adaptability.

### Implications and limitations

China’s universal two-child policy significantly affected every family. The birth of the second child often causes some physical and psychological problems compared to that of the first. To raise second-born children, parents use a lot of time, resources, and attention that were originally solely focused on firstborns. Compared to the parents of two or more children, parents of only one child need to pay special attention to a new type of relationship that emerges after the birth of the second child, Parents should focus on first-born children’s abnormal behaviors and mental health regularly. Those with a withdrawal temperament often have low self-regulation ability. As parenting style is an adjustment factor, parents should adopt individual and differentiated parenting styles. They should focus on communicating with first-born children, showing them more care and understanding. Some parents adopt an authoritarian parenting style toward first-born children because of excessive work or parenting pressure, and solve parenting problems in an abrupt and violent manner. To alleviate the pressure of second-child pregnancy, it is recommended that second-child birth subsidies, specific social care and special treatment for second-child mothers, or reproductive assistance in the community to relieve their economic and work pressure should be provided.

This study has several limitations. First, psychological adjustment questionnaires mainly cover behavioral and emotional problems but neglect interpersonal adaptation (e.g., sibling relationships, parent–child relations). Second, All the data were obtained by parental questionnaires and the measurement of parenting styles only addresses mothers’ perspective. Future studies may try to collect data from multiple informants (e.g., fathers and grandparents) to deepen the current findings.Third, parents' sibling status and their own lack of experience in managing negative emotions related to their sibling's birth were not included in the study. It should be an important measure in futures studies on the subject.

## Data Availability

The data that support the findings of this study are available from the corresponding author upon reasonable request.
